# Severe COVID-19 and its cardiopulmonary effects 6 and 18 months after hospital discharge

**DOI:** 10.3389/fcvm.2024.1366269

**Published:** 2024-03-05

**Authors:** J. H. Niebauer, A. Iscel, S. Schedl, C. Capelle, M. Kahr, S. Schamilow, J. Faltas, M. Srdits, R. Badr-Eslam, M. Lichtenauer, A. Zoufaly, R. Valenta, S. Hoffmann, S. Charwat-Resl, C. Krestan, W. Hitzl, C. Wenisch, D. Bonderman

**Affiliations:** ^1^ Department of Cardiology and Emergency Medicine, Klinik Favoriten, Vienna, Austria; ^2^ Department of Internal Medicine II, Division of Cardiology, AKH Wien, Vienna, Austria; ^3^ Department of Internal Medicine II, Division of Cardiology, Paracelsus Medical University of Salzburg, Salzburg, Austria; ^4^ Department of Infectious Diseases, Klinik Favoriten, Vienna, Austria; ^5^ Faculty of Medicine, Sigmund Freud University, Vienna, Austria; ^6^ Department of Radiology, Klinik Favoriten, Vienna, Austria; ^7^ Research and Innovation Management (RIM), Team Biostatistics and Publication of Clinical Trial Study, Paracelsus Private Medical University, Salzburg, Austria; ^8^ Department of Ophthalmology and Optometry, Paracelsus Medical University, Salzburg, Austria; ^9^ Research Program Experimental Ophthalmology and Glaucoma Research, Paracelsus Medical University, Salzburg, Austria

**Keywords:** SARS-CoV-2, covid-19, long covid, myocarditis, risk factors, long-term impairment, heart, lung

## Abstract

**Introduction:**

SARS-CoV-2 infection affects the cardiopulmonary system in the acute as well as long-term phase. The aim of the present study was to comprehensively assess symptoms and possible long-term impairments 6 and 18 months after hospitalization for severe COVID-19 infection.

**Methods:**

This prospective registry included patients with PCR-confirmed COVID-19 infection requiring hospitalization. Follow-up approximately 6 months post discharge comprised a detailed patient history, clinical examination, transthoracic echocardiography, electrocardiogram, cardiac magnetic resonance imaging (cMRI), chest computed tomography (CT) scan, pulmonary function test (PFT), six-minute walk test (6MWT) and a laboratory panel. At the time of the second follow-up visit at 18 months, patients without pathologic findings during the first study visit were contacted by phone to inquire about the course of their symptoms. In all other patients all initial examinations were repeated.

**Results:**

Two hundred Patients, who were hospitalized for COVID-19, were contacted by phone and were recruited for the study. Due to dropouts the second study visit was performed in 170 patients. A comparison between the two study visits at 6 and 18 months post discharge showed the following results: Six months after discharge, 73% and 18 months after discharge 52% fulfilled the criteria for Long COVID with fatigue being the most common symptom (49%). Echocardiography at 6 months post discharge showed an impaired left ventricular function in 8% of which 80% returned to normal. Six months post discharge, cMRI revealed pericardial effusion in 17% which resolved in 47% of the 15 patients who underwent a control cMRI. Signs of peri- or myocarditis were present in 5% of the patients and were resolved in all 4 patients who attended control studies. At 6 months, chest CT scans identified post-infectious residues in 24%. In the 25 repeated chest CT scans 20% showed full recovery. Length of in-hospital stay was identified as a significant predictor for persisting Long COVID (95% CI: 1.005–1.12, *p* = 0.03).

**Conclusion:**

Comparing 6 to 18 months, the prevalence of Long COVID decreased over time, but a high symptom burden remained. Structural and functional abnormalities were less frequent than the portrayed symptoms, and it thus remains a challenge to substantiate the symptoms.

## 1 Introduction

The severe acute respiratory syndrome coronavirus type 2 (SARS-CoV-2) affects the cardiopulmonary system in the acute as well as in the long-term phase. Therefore, Long COVID syndrome still presents a major challenge more than 3 years after the beginning of the world-wide pandemic. Despite a rapid development and deployment of very effective vaccines, which reduced the prevalence of Long COVID, a vast number of patients continue to suffer from persisting symptoms (1–4).

Previous studies revealed a Long COVID incidence of 60%–70% even 6 months after hospitalization due to severe COVID-19 infection requiring hospitalization, declining, but still remaining high, with 40%–50% after 12 months. Information is scarce how long symptoms persist (5–8). As symptoms are in general very heterogenous in general and include more than 200 different ones, with fatigue being most common (9, 10), it continues to be difficult to substantiate symptoms with respective underlying pathologies (11).

Early studies revealed cardiac involvement in 20%–40% of affected patients in the acute setting with elevated troponin, reduced left ventricular function, myo- and pericarditis being the most prevalent findings (12–16).

Emerging data demonstrate that pathologies identified by cardiac magnetic resonance imaging (cMRI) after COVID-19 infection mostly resolved after 6 months, suggesting long-term recovery (17).

Regarding pulmonary involvement, radiographic changes observed by chest computed tomography (CT) scans were mainly fibrosis and ground glass opacities (18, 19).

These changes were generally considered reversible, but persisted up to 12 months (19).

Whereas recent studies confirmed that neither cMRI nor pulmonary function testing uncovered pathologies explaining the persistent symptom burden, including fatigue (8, 20–22) a recent metanalysis on cardiopulmonary exercise testing revealed that cardiopulmonary exercise capacity testing was impaired in Long COVID patients, suggesting that potential mechanisms might include deconditioning autonomic function, endothelial dysfunction and muscular or mitochondrial pathology (23).

Concerning Long COVID risk factors, studies to date identified in the acute disease phase: high body-mass index (BMI), older age, female gender, combination of five or more symptoms and most strongly the severity of acute infection (6, 24–30). However, up to now, the underlying pathology of Long COVID still remains elusive in many cases (31).

Even though several studies assessed long-term effects of COVID-19, it remains elusive which percentage of patients continue to suffer from cardiopulmonary impairment and how these symptoms evolve over time.

In the present longitudinal cohort study, we provide a comprehensive analysis of symptoms and possible long-term impairment 6 and 18 months after hospitalization and thus, shed light on the underexplored relation between persisting symptoms and potential pathobiological changes of the cardio-respiratory system.

## 2 Materials and methods

### 2.1 Study design and setting

This was a prospective registry, which enrolled 200 consecutive patients with PCR-confirmed COVID19 infection requiring hospital treatment between February 2020 and October 2021. Patients were contacted by phone between July 2020 and April 2022 and were examined during two study visits, 6 and 18 months after hospital discharge in order to assess the presence and evolution of cardiopulmonary long-term sequelae of COVID-19. Both study visits were performed at the Division of Cardiology, Favoriten Clinic, or at the Division of Cardiology, Medical University of Vienna.

A detailed methodology of study-related clinical examinations has been described in our previous publication reporting the results of the first 150 patients at their 6 months follow-up visit (11).

Long COVID was diagnosed in the presence of at least one persisting symptom at the first study visit (6 months after discharge), which had to be related to the acute infection independent from otherwise already underlying, non-COVID-associated organ dysfunction (32, 33).

Our study complied with the Declaration of Helsinki, as well as local laws, and was approved by the local ethics committee (EK 20-153-0720). All study participants gave their written informed consent.

### 2.2 Population

As previously described in greater detail (11), patients had either been hospitalized on a regular ward (91%, *n* = 182) or an intensive care unit (ICU; 9%, *n* = 18) of our dedicated COVID-19 unit. After hospital discharge, patients were contacted in a consecutive manner. Exclusion criteria were age ≤18 years and/or pregnancy.

### 2.3 Study-related clinical examinations

In brief, all participants were examined in a standardized manner and underwent a cardiac and pulmonary workup including clinical assessment, six-minute walk test (6MWT), blood analysis including cardiac biomarkers, such as N-terminal brain natriuretic peptide (NT-proBNP) and troponin T, transthoracic echocardiography (TTE), cMRI, pulmonary function test (PFT) and chest CT scans. For cMRI investigations, a standardized protocol was followed for morphological and functional cardiac evaluation. The left ventricular ejection fraction was calculated from manually corrected endsystolic and enddiastolic endocardial contours in short axis cine loops using dedicated Medis software (Medis Medical Imaging, Leiden, Netherlands).

At the time of the second follow-up visit at 18 months, patients who did not show any abnormalities during the first study visit or refused to return for further follow-up examinations were contacted by phone to inquire about the course of symptoms (*n* = 46). In all other patients with abnormalities during the first study visit, blood analysis, TTE and 6MWT were repeated (*n* = 124). Chest CT scans, cMRI and PFT were only performed in case of pathological findings during the first study visit.

### 2.4 Statistical analysis

Data were tested for consistency and continuous variables for normality. Results from categorical variables are expressed as absolute numbers and percentages, while continuous variables are shown as mean and standard deviations. NT-proBNP and troponin T were log-transformed with base 10. For between group analyses, continuous variables were compared using a bootstrap-t with and without the assumption of variance homogeneity based on 4,000 Monte Carlo simulations. Pearson's chi-square and Fisher's exact test were applied for discrete variables. Univariable logistic regression models were applied to test for independent risk factors for Long COVID, dyspnea and fatigue using asymptotic as well as *p*-values based on Monte Carlo simulation. Corresponding odds ratios with 95% confidence intervals were used to estimate the effect size of each predictor. All reported tests were two-sided, and *p*-values <0.05 were considered statistically significant. All statistical analyses in this report were performed by use of NCSS (NCSS 2022, NCSS, LLC. Kaysville, UT, USA), STATISTICA 13 (Hill, T. & Lewicki, P. Statistics: Methods and Applications. StatSoft, Tulsa, OK, USA) and IBM SPSS (IBM Corp. Armonk, NY, USA) version 26.

## 3 Results

### 3.1 Study participants

**Figure 1:**
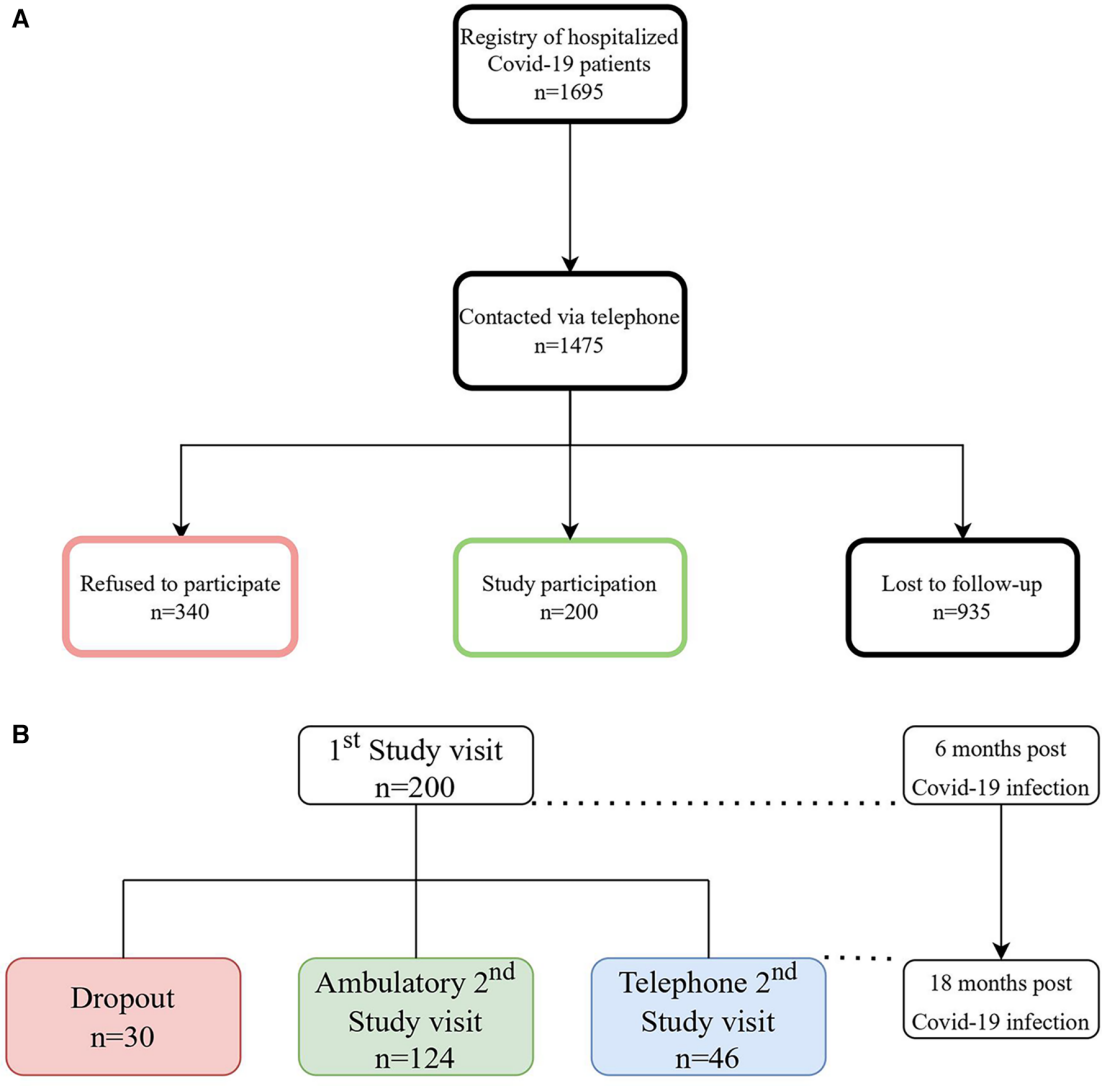
(**A**) Patient flow-chart, *n* = number of patients (**B**) description of the study process of the 1st and 2nd study visit, *n* = number of patients.

A description of the study enrollment procedure is illustrated in Figure 1A. In brief, patients who had been hospitalized between February 2020 and October 2021 were contacted by phone between July 2020 and April 2022. Of the 1,695 consecutive patients who had been listed in our registry of previously hospitalized COVID-19 patients, 1,475 were contacted via phone until 200 patients agreed to participate in the study.

Following the 6-months follow-up visit, a total of 30 patients dropped out as they had either discontinued (5,5%; *n* = 11) the study or had been lost to follow-up (9,5%; *n* = 19) as depicted in Figure 1B.

The 2nd study visit was either carried out in person ambulatory (62%; *n* = 124), for those with abnormal results in at least one of the examinations during the 1st study visit, or via telephone (23%; *n* = 46), for those without abnormalities within the 1st study visit (14%; *n* = 28) or those refusing to come to hospital a second time (9%; *n* = 18).

The first study visit was 6.1 (± 1.7) months and the second 19.1 (± 4.6) months after hospital discharge.

Only 4 of the participants had received COVID-19 vaccination prior to the infection, as the vaccination only became available toward the end of the enrollment period.

### 3.2 Clinical parameters

Detailed patient characteristics 6 months after discharge are depicted in Table 1, with data provided for the entire study population (100%; *n* = 200), symptomatic patients (73%; *n* = 146) and asymptomatic patients (27%; *n* = 54). In brief, the mean duration of COVID-19 hospitalization was 11.0 (±11) days. Patients with a longer in-hospital stay developed Long COVID more frequently (*p* = 0.004).

**Table 1:** Patient characteristics 6 months post discharge and details of in-hospital stay of 200 patients analyzed as total study population and according to symptoms 6 months post COVID-19 infection.

Patient characteristics	Total study population (*n* = 200)	Long COVID (*n* = 146)	Asymptomatic at follow-up (*n* = 54)	*p*-value
Length of in-hospital stay	10.9 (0–84.0)	**12.3 (0–84)**	**6.7 (1–13)**	**0**.004*
Normal ward, %	182 (91.0)	130 (89.0)	52 (96.3)	0.16
Intensive care ward, %	18 (9.0)	16 (11.0)	2 (3.7)	0.16
Age, years	53.3 ± 14.0	54.3 ± 13.9	50.78 ± 14.1	0.47
Female, %	85 (43)	65 (45)	20 (37)	0.34
BMI, kg/m^2^	29.4 ± 5.7	29.3 ± 5.6	29.8 ± 6.2	0.61
SBP, mmHg	136.8 ± 19.3	136.5 ± 18.6	137.8 ± 21.3	0.67
DBP, mmHg	85.6 ± 11.2	85.1 ± 11.3	87.1 ± 10.9	0.31
Heart rate, bpm	71.6 ± 10.7	71.0 ± 10.4	73.2 ± 11.4	0.24
SpO_2_, %	97.8 ± 1.2	**97.6** ± **1.2**	**98.1** ± **1.1**	**0**.039*
Arterial hypertension, %	90 (45.0)	64 (43.8)	26 (48.1)	0.59
Coronary artery disease, %	12 (6.0)	11 (2.7)	1 (1.9)	0.13
preexisting heart failure, %	4 (2.0)	4 (2.8)	0 (0.0)	0.22
Overweight, %	144 (72.0)	103 (72.0)	41 (77.4)	0.45
Diabetes mellitus, %	37 (18.5)	**22 (15.1)**	**15 (27.8)**	**0**.04*
COPD or Asthma, %	18 (9.0)	15 (10.6)	3 (5.6)	0.27
6MWT, m	556.7 ± 99.3	549.6 ± 97.3	577.7 ± 104.1	0.49
Hemoglobin, g/dl	14.3 ± 1.6	14.2 ± 1.5	14.3 ± 2.0	0.77
eGFR, ml/min/L,73 m^2^	85.5 ± 19.6	84.8 ± 20.0	87.5 ± 18.6	0.40
CRP, mg/dl	0.95 ± 3.1	0.8 ± 2.8	1.5 ± 3.8	0.14
NT-proBNP log, pg/ml^a^	4.0 ± 1.2	4.1 ± 1.2	3.8 ± 1.4	0.08
Troponin T log, ng/L^a^	1.8 ± 0.6	1.8 ±0.6	1.8 ± 0.7	0.59
CK, U/L	139.5 ± 95.3	134.2 ± 85.5	153.7 ± 117.4	0.21
Ventilation				
Oxygen, %	80 (40.0)	59 (40.4)	21 (38.9)	0.87
Nasal high flow, %	26 (13.0)	18 (12.3)	8 (14.8)	0.64
NIV, %	3 (1.5)	3 (2.1)	0 (0.0)	0.56
Invasive ventilation, %	15 (7.5)	13 (8.9)	2 (3.7)	0.36

SBP, systolic blood pressure; DBP, diastolic blood pressure; bpm, beats per minute; SpO2, peripheral oxygen saturation; COPD, chronic obstructive pulmonary disease; 6MWT, 6-minute walk test; eGFR, estimated glomerular filtration rate calculated using the Cockroft Gault formula; CRP, C-reactive protein; NT-proBNP, N-terminal pro brain natriuretic peptide; CK, creatine kinase; NIV, noninvasive ventilation. Overweight was defined as a BMI of ≥ 25 kg/m^2^ according to the definition of the World Health Organization. Categorical variables are shown as absolute numbers and percentages, while continuous variables are given as means with standard deviations. *P*-values are based on independent *t*-tests or bootstrap-t tests for continuous variables and Pearson's Chi-Squared or Fisher's Exact Test for discrete variables.

^a^ Log-transformed (base 10).

* Highlights significant *p*-values.

^a^ Bold values represent the statistically significant results.

Mean age of the total study population was 53.3 (±14) years and 85 patients (42.5%) were female. Overweight and a history of arterial hypertension were the most common comorbidities.

Regarding laboratory results, cardiac or inflammatory markers were not elevated 6 months post COVID-19 and no differences with respect to laboratory parameters in Long COVID vs. asymptomatic patients were found (*p* = 0.08, *p* = 0.59, *p* = 0.14).

Patient characteristics of the 170 (85%) patients attending the second study visit are depicted in Table 2. The only significant difference 18 months after hospital discharge between patients suffering from Long COVID and the asymptomatic group was the length of the 6MWT, which had been shorter in the Long COVID group (*p* = 0.003).

**Table 2:** Patient characteristics of 170 patients 18 months post discharge at their second follow-up, analyzed as a total study population as well as subdivided according to symptoms.

Patient characteristics	Total study population (*n* = 170)	Long COVID (*n* = 90)	Asymptomatic at follow-up (*n* = 80)	*p*-value
Length of in-hospital stay	10.7 (0–84)	12.1 (1–84)	9.2 (0–62)	0.09
Normal ward, %	154 (90.6)	80 (88.9)	74 (92.5)	0.42
Intensive care ward, %	16 (9.4)	10 (11.1)	6 (7.5)	0.42
Age, years	53.3 ± 14.0	53.9 ± 13.6	52.4 ± 12.8	0.48
Female, %	67 (39)	39 (43)	28 (35)	0.27
BMI, kg/m^2^	30.24 ± 10.2	30.65 ± 11.9	29.57 ± 6.3	0.58
SBP, mmHg	134.8 ± 20.1	136.0 ± 19.3	132.9 ± ± 21.4	0.48
DBP, mmHg	85.8 ± 14.2	85.3 ± 11.2	86.6 ± 18.4	0.66
Heart rate, bpm	69.9 ± 10.7	69.3 ± 10.1	70.8 ± 11.7	0.48
SpO_2_, %	96.7 ± 10.3	95.9 ± 13.2	97.8 ± 1.9	0.41
6MWT, m	539.8 ± 77.6	**521.0** ± **80.8**	**569.7** ± **62.5**	**0**.003*
Arterial hypertension, %	74 (43.5)	40 (44.4)	34 (42.5)	0.80
Coronary artery disease, %	9 (5.4)	6 (6.8)	3 (3.8)	0.38
Preexisting heart failure, %	4 (2.4)	3 (3.4)	1 (1.3)	0.36
Overweight, %	90 (78.3)	56 (77.8)	34 (79.1)	0.87
Diabetes mellitus, %	31 (18.2)	14 (15.6)	17 (21.3)	0.34
COPD or Asthma, %	14 (8.5)	7 (8.1)	7 (8.9)	0.87
Hemoglobin, g/dl	15.4 ± 11.9	14.3 ± 1.3	17.2 ± 18.8	0.19
eGFR, ml/min/1,73 m^2^	79.2 ± 14.1	77.4 ± 15.4	82.0 ± 11.4	0.11
CRP, mg/dl	2.7 ± 4.1	2.4 ± 3.6	3.2 ± 4.8	0.34
NT-proBNP, pg/ml^a^	3.8 ± 1.2	3.9 ± 1.2	3.6 ± 1.1	0.06
Troponin T, ng/L^a^	1.9 ±0.6	2.01 ± 0.6	1.8 ± 0.4	0.12
CK, U/L	139.8 ± 127.6	123.31 ± 71.9	164.9 ± 180.8	0.08
Ventilation				
Oxygen, %	72 (42.9)	39 (44.3)	33 (41.3)	0.88
Nasal high flow, %	21 (12.5)	10 (11.1)	12 (15.0)	0.50
NIV, %	3 (1.8)	2 (2.3)	1 (1.3)	0.67
Invasive ventilation, %	13 (7.5)	8 (9.1)	5 (6.3)	0.39

SBP, systolic blood pressure; DBP, diastolic blood pressure; bpm, beats per minute; SpO2, peripheral oxygen saturation; 6MWT, 6-minute walk test; COPD, chronic obstructive pulmonary disease; eGFR, estimated glomerular filtration rate calculated using the Cockroft Gault formula; CRP, C-reactive protein; NT-proBNP, N-terminal pro brain natriuretic peptide; CK, creatine kinase; NIV, noninvasive ventilation. Overweight was defined as a BMI of ≥ 25 kg/m^2^ according to the definition of the World Health Organization. Categorical variables are shown as absolute numbers and percentages, while continuous variables are given as means with standard deviations. *P*-values are based on independent *t*-tests or bootstrap-t tests for continuous variables and Pearson's Chi-Squared or Fisher's Exact Test for discrete variables.

^a^ Log-transformed (base 10).

* Highlights significant *p*-values.

^a^ Bold values represent the statistically significant results.

### 3.3 Imaging parameters

**Figure 2:**
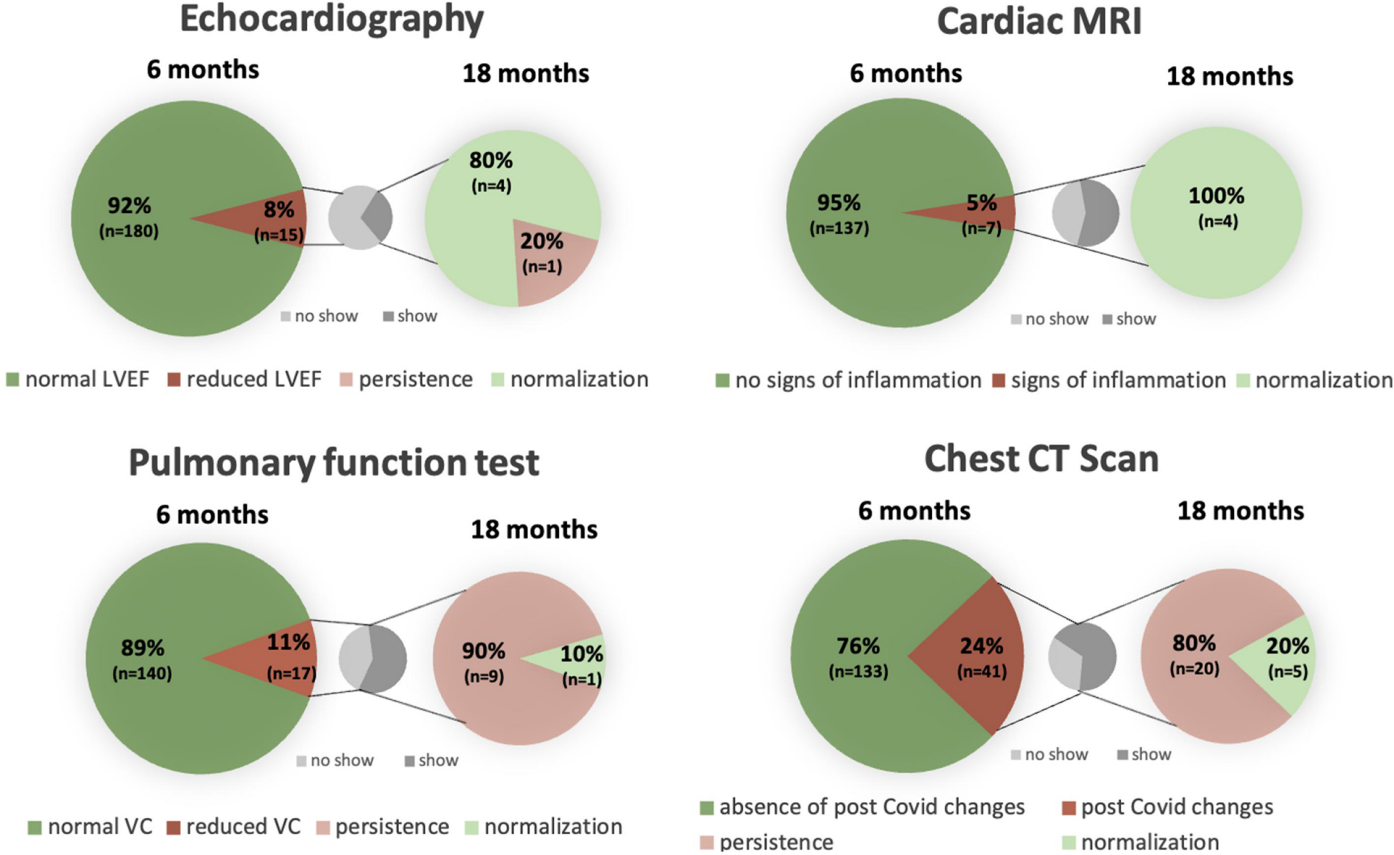
Cardiac and pulmonary structural and functional changes 6 and 18 months post COVID-19 in comparison with a separate graph pointing out the percentage of patients showing up for control after previous abnormalities. LVEF, left ventricular ejection fraction; MRI, magnetic resonance imaging; VC, vital capacity; CT, computed tomography.

Imaging parameters 6 and 18 months post COVID-19 are shown in Figure 2 as well as in Table 3.

**Table 3:** Imaging parameters of the 200 study participants 6 months post COVID-19 infection, analyzed as a total study population as well as subdivided according to symptoms 18 months post COVID-19 infection.

Imaging parameters	Total study population (*n* = 200)	Long COVID (*n* = 146)	Asymptomatic at follow-up (*n* = 54)	*p*-value
Echocardiography				
EF, %	60.3 ± 6.8	60.5 ± 6.5	59.9 ± 7.3	0.98
GLS, %	−18.7 ± 2.9	−18.6 ± 2.7	19.1 ± 3.2	0.37
Diastolic dysfunction, (*n*, %)	9 (4.6)	57 (4.8)	2 (3.8)	0.24
Cardiac MRI				
EF, %	59.2 ± 7.0	59.7 ± 6.8	57.99 ± 7.5	0.35
LGE, (*n*, %)	12 (8.2)	7 (6.5)	5 (12.8)	0.30
Myocarditis/pericarditis, (*n*, %)	7 (4.8)	4 (3.7)	3 (7.7)	0.32
Pericardial effusion, (*n*, %)	27 (19.0)	21 (19.8)	6 (15.4)	0.64
T1 time, ms	1,033.9 ± 39.7	1,033.6 ± 40.2	1,034.9 ± 39.0	0.96
ECV, %	25.6 ± 2.6	25.7 ± 2.6	25.4 ± 2.7	0.50
Chest CT scans	* *	* *	* *	* *
Post COVID changes, (*n*, %)	41 (23.6)	32 (25.2)	9 (19.1)	0.49
Pulmonary function test				
Reduced VC, (*n*, %)	17 (10.8)	10 (8.8)	7 (15.9)	0.20
Reduced FeV1/VC, (*n*, %)	3 (2.0)	2 (1.8)	1 (2.2)	0.86

LVEF, left ventricular ejection fraction; LV-GLS, left ventricular global longitudinal strain measured using speckle tracking imaging; cMRI, cardiac magnetic resonance imaging; LGE, late gadolinium enhancement; ECV, extracellular volume; CT, computed tomography; VC, vital capacity. Categorical variables are shown as absolute numbers and percentages, while continuous variables are given as means with standard deviations. *P*-values are based on independent *t*-tests or bootstrap-t tests for continuous variables and Pearson's Chi-Squared or Fisher's Exact Test for discrete variables.

Six months post COVID-19 infection, impaired left ventricular function in echocardiography was detected in 15 (8%) study participants, of which 5 patients attended their 18-month control echocardiography, where normalization of left ventricular function could be documented in 4 (80%) of these 5 patients. At 6 months, reduced global longitudinal strain (GLS) was measured in 19 (14%) study participants.

cMRI revealed pericardial effusion in 27 (17%) study participants, which resolved after 18 months in 8 (57%) of the 14 patients, who underwent a control cMRI. Signs of peri- or myocarditis were present in 7 (5%) of the patients at 6 months and regressed in all 4 (100%) patients in control studies at 18 months. None of the patients with postinfectious changes had received vaccination before the first study visit.

Pulmonary manifestation at 6 months included reduced vital capacity in 17 (11%) patients in PFT. Values improved in almost half of the patients (47%; *n* = 8) and normalized in 1 of the 10 (10%) patients, who attended the second study visit after 18 months.

At 6 months, chest CT scans identified post-infectious residues in 41 (24%), which mainly included bilateral ground glass opacities, pneumonic consolidation, lymph node enlargement and/or fibrosis. In the 25 repeated chest CT scans 5 (20%) showed full recovery after 18 months. Bilateral consolidations and scarred residues were the most common persisting changes.

Neither a reduced vital capacity in the PFT (*p* = 0.31) nor bilateral changes in the chest CT scans (*p* = 0.54) were associated with persisting dyspnea.

No significant differences in terms of imaging parameters were detected between the Long COVID and the asymptomatic group in the first study visit 6 months as well as in the second study visit 18 months post COVID-19 infection (Tables 3, 4).

**Table 4:** Imaging parameters of the 170 study participants at their second follow-up, analyzed as a total study population as well as subdivided according to symptoms 18 months post COVID-19 infection.

Imaging parameters	Total study population (*n* = 170)	Long COVID (*n* = 90)	Asymptomatic at follow-up (*n* = 80)	*p*-value
Echocardiography				
EF, % (*n* = 113)	62.4 ± 6.0	62.51 ± 6.45	62.30 ± 5.33	0.86
GLS, % (*n* = 61)	−18.7 ± 5.3	−18.52 ± 5.89	19.50 ± 2.62	0.86
Diastolic dysfunction, (*n*, %) (*n* = 110)	3 (2.7)	1 (1.5)	2 (4.5)	0.56
Cardiac MRI				
EF, % (*n* = 22)	58.2 ± 5.7	57.9 ± 4.7	58.4 ± 7.0	0.86
LGE, (n,%) (*n* = 22)	3 (13.6)	1 (8.3)	2 (20.0)	0.45
Myocarditis/pericarditis, (*n*, %) (*n* = 22)	0 (0)	0 (0)	0 (0)	1.0
Pericardial effusion, (*n*, %) (*n* = 22)	7 (31.8)	4 (33.3)	3 (30.0)	1.0
T1 time, ms (*n* = 22)	1,041.0 ± 54.4	1,050.0 ± 41.3	1,031.0 ± 67.8	0.43
ECV, % (*n* = 20)	25.1 ±2.6	25.1 ± 2.5	25.00 ± 2.9	0.91
Chest CT	* *	* *	* *	* *
Post COVID changes, (*n*, %) (*n* = 41)	22 (53.9)	10 (50.0)	11 (61.0)	0.53
Pulmonary function test				
Reduced VC, (*n*, %) (*n* = 31)	11 (35.5)	3 (15.8)	8 (66.7)	0.007
Reduced FeV1/VC (*n*,%)	1 (3.2)	1 (5)	0 (0)	–

LVEF, left ventricular ejection fraction; LV-GLS, left ventricular global longitudinal strain measured using speckle tracking imaging; cMRI, cardiac magnetic resonance imaging; LGE, late gadolinium enhancement; ECV, extracellular volume; CT, computed tomography; VC, vital capacity. Categorical variables are shown as absolute numbers and percentages, while continuous variables are given as means with standard deviations. *P*-values are based on independent *t*-tests or bootstrap-t tests for continuous variables and Pearson's Chi-Squared or Fisher's Exact Test for discrete variables.

### 3.4 COVID-19-related symptoms

**Figure 3:**
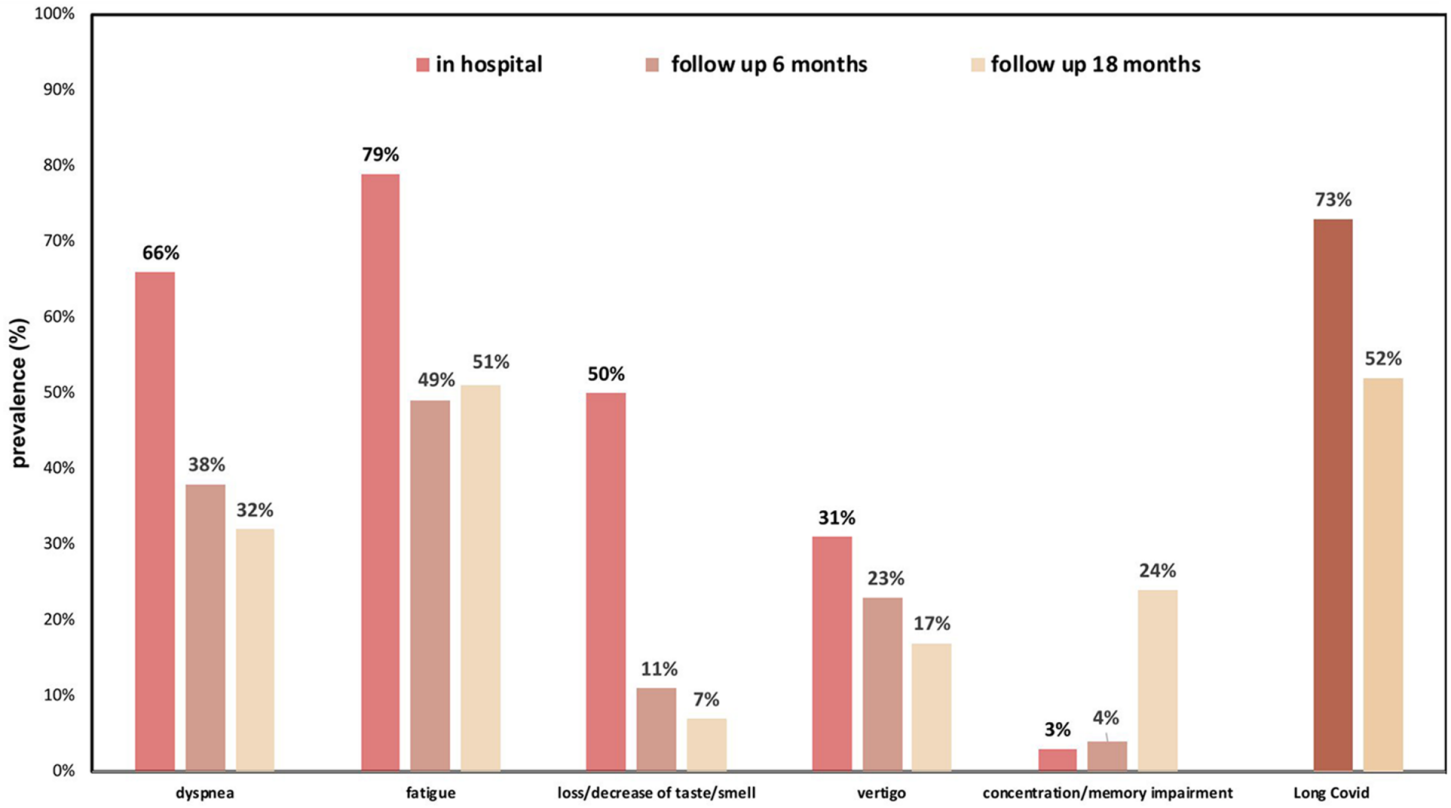
Spectrum of Long COVID symptoms 6 and 18 months post discharge in relation to acute phase symptoms during hospital stay.

A comparison concerning symptoms between the two study visits at 6 and 18 months post discharge showed the following results: Six months after discharge, 73% (*n* = 146) and 18 months after discharge 52% (*n* = 89) still fulfilled criteria for Long COVID with fatigue and exertional dyspnea being the most common symptoms (Figure 3).

Most symptoms were less frequently detected over time except for fatigue and concentration/memory loss, which both increased. Other less frequent symptoms included: chronic head ache, myalgia, palpitations, psychiatric issues and many more.

### 3.5 Long COVID risk factors

The only predictor for persisting Long COVID 6 months post discharge was the length of in-hospital stay [OR 1.14 (95% CI: 1.005–1.12), *p* = 0.03] as an indicator for the severity of the initial disease course (Table 5). No significant risk factors were detected for Long COVID at 18 months (Table 6).

**Table 5:** Risk factors for developing long COVID at 6 months post COVID-19 infection.

Risk factors for long COVID	*p*-value^#^	Odds ratio	95% CI
Length of in-hospital stay	**0** **.** 004*	**1**.**14**	**1.05–1.26**
Admission to intensive care unit	0.13	** **	** **
Age	0.12	1.02	0.995–1.04
Gender	0.34	0.73	0.39–1.39
Overweight	0.45	0.75	0.36–1.58
Previous illness	0.12	1.66	0.88–3.14
NT-proBNP^a^	0.09	1.27	0.97–1.66
Troponin T^a^	0.59	1.16	0.68–1.96
Ventilation			
Oxygen	0.62	1.21	0.56–2.64
Nasal high flow	0.70	0.81	0.28–2.36
NIV	1.0	b)	b)
Intubation	0.14	4.86	0.59–40.3
Pericardial effusion in MRI	0.54	1.35	0.50–3.67
Reduced LVF in echocardiography	0.92	1.07	0.32–3.50
Borderline GLS in echocardiography	0.50	1.40	0.53–3.67
Reduced GLS in echocardiography	0.49	1.52	0.45–5.07
CT post COVID Changes	0.41	1.42	0.62–3.26
Reduced VC	0.21	0.51	0.18–1.45

NT-proBNP, N-terminal pro brain natriuretic peptide; NIV, noninvasive ventilation, MRI, magnetic resonance imaging; LVF, left ventricular ejection fraction; GLS, left ventricular global longitudinal strain; CT, computed tomography; NIV, noninvasive ventilation; VC, vital capacity.

b) Cannot be computed.

^a^ Log (base 10) transformed.

^#^ *p*-values based on univariate logistic regressions.

* Highlights significant *p*-values.

Bold values represent the statistically significant results.

**Table 6:** Risk factors for developing long COVID at 18 months post COVID-19 infection.

Risk factors for long COVID	*p*-value^#^	Odds ratio	95% CI
Length of in-hospital stay	0.12	1.03	1.99–1.06
Admission to intensive care unit	0.54	1.41	0.48–4.14
Age	0.6	1.01	0.98–1.03
Gender	0.28	0.71	0.38–1.32
Overweight	0.74	0.89	0.45–1.78
Previous illness	0.71	1.12	0.60–2.10
NT-proBNP^a^	0.35	1.13	0.88–1.46
Troponin T^a^	0.66	1.13	0.65–1.95
Ventilation			
Oxygen	0.71	1.14	0.57–2.28
Nasal high flow	0.53	0.73	0.27–1.98
NIV	0.60	1.93	0.17–22.5
Intubation	0.49	1.55	0.45–5.28
Pericardial effusion in MRI	0.97	0.98	0.41–2.37
Reduced LVF in echocardiography	0.18	2.53	0.65–9.90
Borderline GLS in echocardiography	0.11	2.07	0.84–5.11
Reduced GLS in echocardiography	0.81	1.15	0.37–3.62
CT post COVID Changes	0.65	0.84	0.39–1.80
Reduced VC	0.32	0.55	0.17–1.77

NT-proBNP, N-terminal pro brain natriuretic peptide; NIV, noninvasive ventilation MRI, magnetic resonance imaging; LVF, left ventricular ejection fraction; GLS, left ventricular global longitudinal strain; CT, computed tomography; VC, vital capacity.

^a^ Log (base 10) transformed.

^#^ *p*-values based on univariate logistic regressions.

Bold values represent the statistically significant results.

Testing for specific symptoms, six months after discharge, length of in-hospital stay was a risk factor for fatigue [OR 1.06 (95% CI 1.008–1.107), *p* = 0.021] as well as for persisting dyspnea [OR 1.069 (95% CI 1.035–1.16), *p* = 0.001]. In addition, overweight [OR 2.33 (95% CI 1.15–4.74), *p* = 0.003] and reduced GLS in echocardiography were predictive for exertional dyspnea [OR 3.69 (95% CI 1.3–10.47), *p* = 0.005, Table 7].

**Table 7:** Risk factors for developing fatigue or exertional dyspnea 6 months after hospital admission due to COVID-19.

Risk factors for fatigue	*p*-value^#^	Odds ratio	95% CI
Length of in-hospital stay	**0** **.** 021*	**1**.**06**	**1.008**–**1.107**
Admission to intensive care unit	0.26	1.78	0.66–4.78
Age	0.67	1.01	0.99–1.03
Gender	0.39	0.78	0.46–1.37
Overweight	0.80	0.92	0.49–1.74
Previous illness	0.30	1.36	0.76–2.41
NT-proBNP^a^	0.67	1.05	0.84–1.32
Troponin T^a^	0.75	0.93	0.57–1.49
Ventilation	** **	** **	** **
Oxygen	0.96	0.98	0.52–1.85
Nasal high flow	0.53	1.33	0.55–3.26
NIV	0.65	0.57	0.05–6.57
Invasive ventilation	0.35	1.71	0.55–5.30
Pericardial effusion in MRI	0.10	0.48	0.19–1.14
Reduced LVF in echocardiography	0.94	0.96	0.33–2.76
Borderline GLS in echocardiography	0.21	1.71	0.75–3.94
Reduced GLS in echocardiography	0.20	1.94	0.71–5.34
Diastolic dysfunction	0.77	0.82	0.21–3.14
CT post COVID Changes	0.76	1.12	0.55–2.25
Reduced VC	0.56	0.74	0.27–2.06
Risk factors for Exertional dyspnea	*p*-value^#^	Odds ratio	95% CI
Length of in-hospital stay	**0**.001*	**1**.**096**	**1.035**–**1.16**
Admission to intensive care unit	0.10	2.18	0.82–5.79
Age	0.25	1.01	0.99–1.03
Gender	0.65	0.88	0.49–1.56
Overweight	**0**.003*	**2**.**33**	**1.15**–**4.74**
Previous illness	0.41	1.28	0.71–2.32
NT-proBNP^a^	0.75	1.04	0.82–0.132
Troponin T^a^	0.64	0.88	0.54–1.44
Ventilation			
Oxygen	0.58	1.21	0.62–2.35
Nasal high flow	0.051	2.48	0.99–6.17
NIV	0.96	1.06	0.09–12.3
Invasive ventilation	0.12	2.43	0.09–12.3
Pericardial effusion in MRI	0.24	1.66	0.71–3.90
Reduced LVF in echocardiography	0.074	2.67	0.91–7.84
Borderline GLS in echocardiography	0.33	0.63	0–24–1.64
Reduced GLS in echocardiography	**0**.005*	**3**.**69**	**1.3**–**10.47**
Diastolic dysfunction	0.09	3.41	0.83–14.1
CT post COVID Changes	0.49	1.28	0.63–2.61
Reduced VC	0.54	1.37	0.50–0.378

NT-proBNP, N-terminal pro brain natriuretic peptide; NIV, noninvasive ventilation, LVF, left ventricular ejection fraction; GLS, left ventricular global longitudinal strain; CT, computed tomography; VC, vital capacity.

^a^ Log (base 10) transformed.

^#^ *p*-values based on univariate logistic regressions.

* Highlights significant *p*-values.

Bold values represent the statistically significant results.

### 3.6 Subgroup analysis normal ward vs. ICU

A subgroup analysis comparing normal ward and ICU patients revealed differences in the 6-minute walk distance (normal ward: 520.6 ± 102.28 m vs. ICU: 434.54 ± 135.45 m, *p* = 0.006, Table 8) at 6 months as well as at 18 months (normal ward: 546.1 ± 73.22 m vs. ICU: 423.6 ± 72.06 m, *p* = 0.001, Table 9). No between-group differences were encountered with respect to laboratory parameters at 6 or 18 months after discharge (Tables 8, 9).

**Table 8:** Patient characteristics of 200 patients at their first follow-up 6 months post discharge after COVID-19 infection according to hospital admission to either normal ward or ICU.

Patient characteristics	Normal ward (*n* = 182, 90%)	Intensive care unit (*n* = 18, 10%)	*p*-value
length of in-hospital stay	**8.62 ** **± ** **5.16**	**34.44 **± **22.05**	**0**.0005*
Age, years	53.06 ± 14.3	55.56 ± 10.86	0.47
Female, %	79 (43%)	6 (33%)	0.17
BMI, kg/m^2^	29.16 ± 5.53	31.16 ± 7.02	0.41
SBP, mmHg	136.3 ± 19.14	140.93 ± 20.63	0.41
DBP, mmHg	85.47 ± 10.79	85.64 ± 15.89	0.96
Heart rate, bpm	71.28 ± 10.45	71.21 ± 9.12	0.99
SpO_2_, %	97.85 ± 1.09	96.6 ± 1.9	0.095
Arterial hypertension, %	80 (44%)	9 (50%)	0.80
Coronary artery disease 60, %	9 (5%)	3 (17%)	0.08
preexisting heart failure 59, %	**1 (0.5%)**	**3 (16.7%)**	**0**.002*
Overweight, %	128 (73%)	14 (78%)	0.78
Diabetes mellitus, %	31 (17%)	5 (28%)	0.33
COPD or Asthma, %	17 (10%)	1 (5.6%)	1.0
6MWT, m	**520.6 **± **102.28**	**434.54 **± **135.45**	**0**.006*
Hemoglobin, g/dl	15.1 ± 10.4	13.7 ± 1.87	0.077
eGFR, ml/min/1,73m^2^	86.29 ± 19.7	79.51 ± 18.6	0.19
CRP, mg/dl	0.82 ± 3.04	1.66 ± 3.09	0.14
NT-proBNP log, pg/ml^#^	3.97 ± 1.15	4.67 ± 1.68	0.11
Troponin T log, ng/L^#^	142 ± 95.13	116.19 ± 101.8	0.43
CK, U/L	8.62 ± 5.16	34.44 ± 22.05	0.32

SBP, systolic blood pressure; DBP, diastolic blood pressure; bpm, beats per minute; SpO2, peripheral oxygen saturation; COPD, chronic obstructive pulmonary disease; 6MWT, 6-minute walk test; eGFR, estimated glomerular filtration rate calculated using the Cockroft Gault formula; CRP, C-reactive protein; NT-proBNP, N-terminal pro brain natriuretic peptide; CK, creatine kinase. Overweight was defined as a BMI of ≥ 25 kg/m^2^ according to the definition of the World Health Organization. Categorical variables are shown as absolute numbers and percentages, while continuous variables are given as means with standard deviations. *P*-values are based on independent *t*-tests or bootstrap-*t* tests for continuous variables and Pearson's Chi-Squared or Fisher's Exact Test for discrete variables.

^#^ Log-transformed (base 10).

* Highlights significant *p*-values.

Bold values represent the statistically significant results.

**Table 9:** Patient characteristics of 170 patients at their second follow-up 18 months post discharge after COVID-19 infection according to hospital admission to either normal ward or ICU.

Patient characteristics	Normal ward (*n* = 154)	Intensive care unit (*n* = 16)	*p*-value
SBP, mmHg	134.54 ± 19.75	141.17 ± 26.48	0.45
DBP, mmHg	85.9 ± 14.09	85.17 ± 18.64	0.89
Heart rate, bpm	69.85 ± 10.44	66.38 ± 9.86	0.34
SpO_2_, %	96.62 ± 10.62	97 ± 1.41	0.92
6MWT, m	**546.1 ** **± ** **73.22**	**423.6 **± **72.06**	**0**.001*
Hemoglobin, g/dl	15.61 ± 12.55	14.03 ± 1.57	0.17
eGFR, ml/min/1,73m^2^	79.55 ± 13.90	75.04 ± 15.93	0.34
CRP, mg/dl	2.69 ± 4.2	2.96 ± 3.88	0.83
NT-proBNP, pg/ml^#^	3.75 ± 1.08	4.64 ± 1.8	0.12
Troponin T, ng/L^#^	1.92 ± 0.53	2.15 ± 0.92	0.43
CK, U/L	**145.39 **± **134.14**	**98.15 **± **42.32**	**0**.024*

SBP, systolic blood pressure; DBP, diastolic blood pressure; bpm, beats per minute; SpO2, peripheral oxygen saturation; 6MWT, 6-minute walk test; COPD, chronic obstructive pulmonary disease; eGFR, estimated glomerular filtration rate calculated using the Cockroft Gault formula; CRP, C-reactive protein; NT-proBNP, N-terminal pro brain natriuretic peptide; CK, creatine kinase. Overweight was defined as a BMI of ≥ 25 kg/m^2^ according to the definition of the World Health Organization. Categorical variables are shown as absolute numbers and percentages, while continuous variables are given as means with standard deviations. *P*-values are based on independent *t*-tests or bootstrap-t tests for continuous variables and Pearson's Chi-Squared or Fisher's Exact Test for discrete variables.

^#^ Log-transformed (base 10).

* Highlights significant *p*-values.

Bold values represent the statistically significant results.

Regarding imaging parameters 6 months after discharge, GLS was significantly more reduced (*p* = 0.033, Table 10) and post-COVID CT changes were more frequent in patients with former ICU hospitalization (*p* = 0.017, Table 10). No significant differences with regards to imaging were found after 18 months between normal ward and ICU patients (Table 11).

**Table 10:** Imaging parameters of the 200 study participants 6 months post COVID-19 infection according to hospital admission to either normal ward or ICU.

Imaging parameters	Normal ward (*n* = 182)	Intensive care unit (*n* = 18)	*p*-value
Echocardiography			
EF, %	60.73 ± 6.22	58.24 ± 7.04	0.12
Normal/intermediate/reduced GLS, (*n*,%)	**81 (65)/27 (22)/16 (13)**	**2 (22)/4 (44)/3 (33)**	**0**.033*
Diastolic dysfunction, (%)	96/0/4	94/0/6	0.95
Cardiac MRI			
EF, %	59.68 ± 6.68	55.09 ± 8.84	1.00
LGE, (*n*,%)	11 (8.1)	1 (9)	1.0
Myocarditis/pericarditis, (*n*,%)	7 (5.3)	0 (0)	1.0
Pericardial effusion, (*n*,%)	25 (18.7)	3 (27.3)	0.44
Chest CT scans			
Post COVID changes, (*n*,%)	**32 (20.1)**	**9 (60)**	**0**.017*
Pulmonary function test			
Reduced VC, (*n*,%)	13 (9.2)	4 (26.7)	0.061

LVEF, left ventricular ejection fraction; LV-GLS, left ventricular global longitudinal strain measured using speckle tracking imaging; cMRI, cardiac magnetic resonance imaging; LGE, late gadolinium enhancement; ECV, extracellular volume; CT, computed tomography; VC, vital capacity. Categorical variables are shown as absolute numbers and percentages, while continuous variables are given as means with standard deviations. *P*-values are based on independent *t*-tests or bootstrap-t tests for continuous variables and Pearson's Chi-Squared or Fisher's Exact Test for discrete variables.

* Highlights significant *p*-values.

Bold values represent the statistically significant results.

**Table 11:** Imaging parameters of the 200 study participants 18 months post COVID-19 infection according to hospital admission to either normal ward or ICU.

Imaging parameters	Normal ward (*n* = 154)	Intensive care unit (*n* = 16)	*p*-value
Echocardiography			
EF, %	62.55 ± 6.2	61.45 ± 4.34	0.57
Normal/intermediate/reduced GLS, (*n*,%)	42 (75)/10 (18)/4 (7)	3 (60)/2 (40)/0 (0)	0.44
Diastolic dysfunction, (*n*,%)	3 (3)	0 (0)	1.0
Cardiac MRI			
EF, %	57.92 ± 5.96	59 ± 0	0.86
LGE, (*n*,%)	3 (15)	0 (0)	1.0
Myocarditis/pericarditis, (*n*,%)	2 (10)	0 (0)	1.0
Pericardial effusion, (*n*,%)	6 (31.6)	1 (100)	0.35
Chest CT scans	* *	* *	* *
Post COVID changes, (*n*,%)	16 (52)	5 (71)	0.43
Pulmonary function test			
Reduced VC, (*n*,%)	8 (32)	3 (50)	0.64

LVEF, left ventricular ejection fraction; LV-GLS, left ventricular global longitudinal strain measured using speckle tracking imaging; cMRI, cardiac magnetic resonance imaging; LGE, late gadolinium enhancement; ECV, extracellular volume; CT, computed tomography; VC, vital capacity. Categorical variables are shown as absolute numbers and percentages, while continuous variables are given as means with standard deviations. *P*-values are based on independent *t*-tests or bootstrap-t tests for continuous variables and Pearson's Chi-Squared or Fisher's Exact Test for discrete variables.

## 4 Discussion

The purpose of this study was to deliver a comprehensive report on the prevalence of symptoms and possible cardio-pulmonary long-term impairments 6 and 18 months after severe COVID-19. The five major findings are:

Firstly, cardiac manifestations have been documented in both TTE and cMRI and mostly resolved over time. In TTE, a reduced left ventricular function was detected in 8%, almost matching the 10% of a previous study of only symptomatic patients 5 months after infection (34). In our study, 80% normalized after 18 months.

Since cMRI is a valuable tool in assessing cardiac involvement and it was thus also used in our study, we were able to detect traces of pericardial effusion in 17% of mostly asymptomatic patients. The reported prevalence in other studies varied between 4.6% as an incidental finding in patients with COVID-19 of mild severity (35) and 90.7% (36) in critically ill patients. Follow-up cMRIs showed resolution of pericardial effusion in more than half of our patients. In accordance with a previous report, we speculate that also in our study pericardial effusion was rather caused by inflammation than infection, as effusions in the respective study were commonly found to be sterile (37). As our first visit was scheduled 6 months after discharge, and a similar study observed that cardiac manifestations of COVID-19 mostly resolved within that timeframe (17), it can be speculated that the rate of cardiac manifestations in our study was actually higher than before reported in similar studies. Furthermore, the incidence of pericardial effusions in our study is lower compared to other studies (36), most likely due to a longer time period between the index infection and the cMRI. The absence of inflammatory changes in the pericardium at 6 months in patients presenting with a pericardial effusion is likely attributable to the putative recovery since the index hospitalization and we interpret the pericardial effusions as detecting the remnants of previous inflammation. This would explain the lacking inflammatory changes on the MRI scans with normal inflammatory parameters in the laboratory panel.

Since we performed cMRIs 6 and 18 months after discharge, we were able to distinguish between cardiac manifestations secondary to COVID-19 and preexisting pathologies.

In a recent international, retrospective, observational study, acute severe COVID-19 myocarditis has been reported to be rather rare with 2.4 “definite/probable” cases per 1,000 hospitalizations increasing to 4.1 when also “possible” cases were considered (38). In our cohort, however, results indicate a higher incidence as signs of peri- or myocarditis were present in 5% of the first cMRI after 6 months, mostly subclinical though, and fully regressed in all follow-up cMRIs. This full resolution of signs of myocarditis also corroborates our clinical findings of complete recovery and supports a causal relationship with COVID-19 rather than coincidental underlying causes. Nonetheless, the exact prevalence of cardiovascular involvement remains unknown but is likely to be underestimated as studies vary regarding their patient inclusion criteria, MRI protocols and follow-up time frames (36).

Secondly, concerning pulmonary manifestation, chest CT scans 6 months post hospital discharge showed postinfectious changes in one fourth of the patients with mainly bilateral ground glass opacities. Follow-up CTs revealed transformation to bilateral consolidations and scarred residues. Although it had been reported that such changes were generally reversible within 12 months (19), in our study, only 23% of our affected patients showed full resolution after 18 months. In keeping with findings from another study, this was not accompanied by pulmonary functional impairment like early exertional dyspnea (39). Taken together, cardiac sequelae of COVID-19 seem to resolve over time, while pulmonary manifestations seem to last longer or be persistent.

Thirdly, six months after hospital discharge, the majority of patients still suffered from at least one symptom and 73% fulfilled the criteria for Long COVID, decreasing to 52% after 18 months. The symptom burden remained high throughout the study course with fatigue and exertional dyspnea being the most common symptoms at both visits, matching findings from other studies (7, 9, 10, 40).

Previous literature reports a 60%–70% incidence of Long COVID 6 months after hospitalization due to severe COVID-19 infection, declining, but still remaining high, with 40%–50% after 12 months (3–6). Given the low number of patients requiring admission to the ICU contrasted by a relatively high number of Long COVID symptoms, this suggests that even patients with a relatively mild disease course may develop Long COVID.

Symptoms decreased over time (7), and as in our study, fatigue and memory impairment were among the most prevalent symptoms.

Fourthly, the only risk factor being predictive for Long COVID 6 months after discharge was the length of the initial hospital stay representing the severity of disease. This has been supported by a recent study from Spain with a higher percentage of Long COVID in patients with previous hospitalization (40, 41). This, however, was only predictive for Long COVID at 6 but not 18 months after discharge in our study, which might be explained by fewer abnormalities as well as a smaller number of affected patients throughout the study.

Risk factors for Long COVID differ considerably among published studies, due to cohorts being rather heterogenous. Most often high body-mass index (BMI), older age, female sex, combination of five or more symptoms and/or severity of acute infection (6, 24–30) were mentioned. None of these, however, were predictive in our study, where length of stay, as mentioned above, was the only predictive variable, which is indirectly correlated to severity of disease.

Fifthly, with regards to the persistence of symptoms, exertional dyspnea could not be substantiated by diagnostic or clinical findings, whereas overweight and a reduced GLS were predictive for exertional dyspnea.

We could not identify any specific clinical manifestation that could have served as a plausible explanation for the leading symptom fatigue, which clinically resembles chronic fatigue syndrome. However, as with other symptoms reported in our study, length of the initial hospital stay and thus a marker of the severity of the disease, was the only predictive risk factor. Recent studies confirm that neither cMRI, chest CT scan nor pulmonary function uncovered pathologies explaining persistent symptom burden, including fatigue (8, 20–22).

Regarding the management of symptoms, existing evidence is limited to exercise training rehabilitation, which improved dyspnea in Long COVID (42). Initial research mainly focused on symptom-centered treatments that included naltrexone against neuro-inflammation, beta blockers for postural tachycardia syndrome, antihistamines and/or intravenous immunoglobulins for immune dysfunction and cognitive pacing for cognitive dysfunction. Current research focuses, among others, on further treatment strategies for Long COVID such as elimination of autoantibodies (31).

### 4.1 Limitations

Firstly, because a relatively large number of registry patients had either been lost to follow-up (*n* = 935), refused to participate (*n* = 340) before inclusion into the study or dropped out (*n* = 30) during the course of the study, an inherent bias of the reported results cannot be excluded.

Therefore, secondly, an even larger sample size would have been favorable. Adherence to control examinations could have been better, but may partially be explained by the fact that persisting symptoms and abnormalities documented during the first study visit were rather minor and possibly disappeared over time.

Thirdly, a subgroup analysis comparing vaccinated and unvaccinated patients was not feasible due to the limited number of individuals who had been vaccinated (*n* = 4) before the COVID-19 infection.

Fourthly, with respect to diagnostics in terms of the laboratory panel, only leucocytes and C-reactive peptide had been measured. Neither cytokines nor other inflammatory parameters, which could have contributed to the pathogenesis of Long Covid, have been measured in the present study.

Fifthly, even though the 6MWT was significantly reduced in the Long COVID group, this test has not been validated for prognostic and pathophysiological consequences in Long COVID patients so far. A better tool for understanding the etiology of the symptoms would have been the cardiopulmonary exercise testing, which should be carried out in future studies.

### 4.2 Conclusion

During the course of our study, patients previously treated in-hospital for severe COVID-19 infection showed a decrease in the prevalence of Long COVID symptoms over time. Nonetheless, even 18 months after hospital discharge still a high symptom burden remained. Also, structural and functional abnormalities documented 6 months after discharge were less frequent after 18 months. Interestingly, we found no correlation between symptoms and structural and/or functional abnormalities, so it remains a challenge to substantiate these symptoms. This raises the question whether immunological factors (43) could contribute to the Long COVID symptoms, which has not been examined in the present study. Our study also revealed that the only significant risk factor for developing Long COVID was the initial length of in-hospital stay, which serves as a marker of disease severity.

In our cohort of patients that previously suffered from severe COVID-19 infection, only few showed abnormalities in cardiopulmonary examinations. In fact, very few patients had cardiac involvement, with long-term effects being rather rare and regressing in most over time.

## Data Availability

The raw data supporting the conclusions of this article will be made available by the authors, without undue reservation.

## References

[1] Sewanan LR Clerkin KJ Tucker NR Tsai EJ. How does COVID-19 affect the heart? Curr Cardiol Rep. (2023) 25:171–84. 10.1007/s11886-023-01841-6 36897483 PMC9999058

[2] Rivera-Izquierdo M Lainez-Ramos-Bossini AJ de Alba IG-F Ortiz-González-Serna R Serrano-Ortiz A Fernández-Martínez NF Long COVID 12 months after discharge: persistent symptoms in patients hospitalised due to COVID-19 and patients hospitalised due to other causes—a multicentre cohort study. BMC Med. (2022) 20:92. 10.1186/s12916-022-02292-6 35193574 PMC8863509

[3] Watanabe A Iwagami M Yasuhara J Takagi H Kuno T. Protective effect of COVID-19 vaccination against long COVID syndrome: a systematic review and meta-analysis. Vaccine. (2023) 41:1783–90. 10.1016/j.vaccine.2023.02.008 36774332 PMC9905096

[4] Ceban F Kulzhabayeva D Rodrigues NB Di Vincenzo JD Gill H Subramaniapillai M COVID-19 vaccination for the prevention and treatment of long COVID: a systematic review and meta-analysis. Brain Behav Immun. (2023) 111:211–29. 10.1016/j.bbi.2023.03.022 36990297 PMC10067136

[5] Fumagalli C Zocchi C Tassetti L Silverii MV Amato C Livi L Factors associated with persistence of symptoms 1 year after COVID-19: a longitudinal, prospective phone-based interview follow-up cohort study. Eur J Intern Med. (2022) 97:36–41. 10.1016/j.ejim.2021.11.018 34903448 PMC8648678

[6] Taquet M Dercon Q Luciano S Geddes JR Husain M Harrison PJ. Incidence, co-occurrence, and evolution of long-COVID features: a 6-month retrospective cohort study of 273,618 survivors of COVID-19. PLoS Med. (2021) 18:e1003773. 10.1371/journal.pmed.1003773 34582441 PMC8478214

[7] Pazukhina E Andreeva M Spiridonova E Bobkova P Shikhaleva A El-Taravi Y Prevalence and risk factors of post-COVID-19 condition in adults and children at 6 and 12 months after hospital discharge: a prospective, cohort study in Moscow (StopCOVID). BMC Med. (2022) 20:244. 10.1186/s12916-022-02448-4 35794549 PMC9257572

[8] Huang L Yao Q Gu X Wang O Ren L Wang Y 1-year outcomes in hospital survivors with COVID-19: a longitudinal cohort study. Lancet. (2021) 398:747–58. 10.1016/S0140-6736(21)01755-4 34454673 PMC8389999

[9] Gyöngyösi M Alcaide P Asselbergs FW Brundel B Camici GG da Costa Martins P Long COVID and the cardiovascular system—elucidating causes and cellular mechanisms in order to develop targeted diagnostic and therapeutic strategies: a joint scientific statement of the ESC working groups on cellular biology of the heart and myocardial and pericardial diseases. Cardiovasc Res. (2023) 119:336–56. 10.1093/cvr/cvac115 35875883 PMC9384470

[10] Maestre-Muñiz MM Arias á Mata-Vázquez E Martín-Toledano M López-Larramon G Ruiz-Chicote AM Long-term outcomes of patients with coronavirus disease 2019 at one year after hospital discharge. J Clin Med. (2021) 10:2945. 10.3390/jcm10132945 34209085 PMC8269002

[11] Niebauer JH Binder-Rodriguez C Iscel A Schedl S Capelle C Kahr M Cardiopulmonary long-term sequelae in patients after severe COVID-19 disease. J Clin Med. (2023) 12:1536. 10.3390/jcm12041536 36836071 PMC9959779

[12] Friedrich MG Cooper LT. What we (don’t) know about myocardial injury after COVID-19. Eur Heart J. (2021) 42:1879–82. 10.1093/eurheartj/ehab145 33713116 PMC8108615

[13] Guo T Fan Y Chen M Wu X Zhang L He T Cardiovascular implications of fatal outcomes of patients with coronavirus disease 2019 (COVID-19). JAMA Cardiol. (2020) 5:811. 10.1001/jamacardio.2020.1017 32219356 PMC7101506

[14] Goudot G Chocron R Augy J-L Gendron N Khider L Debuc B Predictive factor for COVID-19 worsening: insights for high-sensitivity troponin and D-dimer and correlation with right ventricular afterload. Front Med (Lausanne). (2020) 7:8. 10.3389/fmed.2020.586307 33282891 PMC7689153

[15] Shi S Qin M Shen B Cai Y Liu T Yang F Association of cardiac injury with mortality in hospitalized patients with COVID-19 in Wuhan. China. JAMA Cardiol. (2020) 5:802. 10.1001/jamacardio.2020.0950 32211816 PMC7097841

[16] Wang L He W Yu X Hu D Bao M Liu H Coronavirus disease 2019 in elderly patients: characteristics and prognostic factors based on 4-week follow-up. Journal of Infection. (2020) 80:639–45. 10.1016/j.jinf.2020.03.019 32240670 PMC7118526

[17] Cassar MP Tunnicliffe EM Petousi N Lewandowski AJ Xie C Mahmod M Symptom persistence despite improvement in cardiopulmonary health – insights from longitudinal CMR, CPET and lung function testing post-COVID-19. EClinicalMedicine. (2021) 41:101159. 10.1016/j.eclinm.2021.101159 34693230 PMC8527025

[18] Montani D Savale L Noel N Meyrignac O Colle R Gasnier M Post-acute COVID-19 syndrome. Eur Respir Rev. (2022) 31:210185. 10.1183/16000617.0185-2021 35264409 PMC8924706

[19] Wu X Liu X Zhou Y Yu H Li R Zhan Q 3-month, 6-month, 9-month, and 12-month respiratory outcomes in patients following COVID-19-related hospitalisation: a prospective study. Lancet Respir Med. (2021) 9:747–54. 10.1016/S2213-2600(21)00174-0 33964245 PMC8099316

[20] Raman B Bluemke DA Lüscher TF Neubauer S. Long COVID: post-acute sequelae of COVID-19 with a cardiovascular focus. Eur Heart J. (2022) 43:1157–72. 10.1093/eurheartj/ehac031 35176758 PMC8903393

[21] Maiese A Frati P Del Duca F Santoro P Manetti AC La Russa R Myocardial pathology in COVID-19-associated cardiac injury: a systematic review. Diagnostics (Basel). (2021) 11:9–11. 10.3390/diagnostics11091647 PMC8472043 34573988

[22] Kersten J Wolf A Hoyo L Hüll E Tadic M Andreβ S Symptom burden correlates to impairment of diffusion capacity and exercise intolerance in long COVID patients. Sci Rep. (2022) 12:8801. 10.1038/s41598-022-12839-5 35614108 PMC9130688

[23] Durstenfeld MS Sun K Tahir P Peluso MJ Deeks SG Aras MA Use of cardiopulmonary exercise testing to evaluate long COVID-19 symptoms in adults. JAMA Netw Open. (2022) 5:e2236057. 10.1001/jamanetworkopen.2022.36057 36223120 PMC9557896

[24] Förster C Colombo MG Wetzel A-J Martus P Joos S. Persisting symptoms after COVID-19. Dtsch Arztebl Int. (2022) 199:167. 10.3238/arztebl.m2022.0147 PMC9215272 35236547

[25] Jacob L Koyanagi A Smith L Tanislav C Konrad M van der Beck S Prevalence of, and factors associated with, long-term COVID-19 sick leave in working-age patients followed in general practices in Germany. Int J Infect Dis. (2021) 109:203–8. 10.1016/j.ijid.2021.06.063 34224870 PMC8922990

[26] Sudre CH Murray B Varsavsky T Graham MS Penfold RS Bowyer RC Attributes and predictors of long COVID. Nat Med. (2021) 27:626–31. 10.1038/s41591-021-01292-y 33692530 PMC7611399

[27] Yong SJ. Long COVID or post-COVID-19 syndrome: putative pathophysiology, risk factors, and treatments. Infect Dis. (2021) 53:737–54. 10.1080/23744235.2021.1924397 PMC8146298 34024217

[28] Soriano JB Murthy S Marshall JC Relan P Diaz JV A clinical case definition of post-COVID-19 condition by a delphi consensus. Lancet Infect Dis. (2022) 22:e102–7. 10.1016/S1473-3099(21)00703-9 34951953 PMC8691845

[29] Crook H Raza S Nowell J Young M Edison P. Long COVID—mechanisms, risk factors, and management. BMJ. (2021) 374:n1648. 10.1136/bmj.n1648 34312178

[30] Augustin M Schommers P Stecher M Dewald F Gieselmann L Gruell H Post-COVID syndrome in non-hospitalised patients with COVID-19: a longitudinal prospective cohort study. Lancet Reg Health Eur. (2021) 6:100122. 10.1016/j.lanepe.2021.100122 34027514 PMC8129613

[31] Davis HE McCorkell L Vogel JM Topol EJ. Long COVID: major findings, mechanisms and recommendations. Nat Rev Microbiol. (2023) 21:133–46. 10.1038/s41579-022-00846-2 36639608 PMC9839201

[32] Sivan M Taylor S. NICE guideline on long COVID. Br Med J. (2020) 371:m4938. 10.1136/bmj.m4938 33361141

[33] Koczulla A Ankermann T Behrends U. *S1-Leitlinie Long/ Post-COVID*. AWMF online (2022). (accessed October 5, 2023).

[34] Pelà G Goldoni M Cavalli C Perrino F Tagliaferri S Frizelli A Long-term cardiac sequelae in patients referred into a diagnostic post-COVID-19 pathway: the different impacts on the right and left ventricles. Diagnostics. (2021) 11:2059. 10.3390/diagnostics11112059 34829406 PMC8623572

[35] Al-Tarbsheh AH Leamon A Chong WH Chung JK Oweis J Vamshek S Pericardial effusion in COVID-19 patients. Am J Med Sci. (2022) 364:129–30. 10.1016/j.amjms.2022.01.024 35276074 PMC9110270

[36] Liu Y Xie J Gao P Tian R Qian H Guo F Swollen heart in COVID-19 patients who progress to critical illness: a perspective from echo-cardiologists. ESC Heart Fail. (2020) 7:3621–32. 10.1002/ehf2.12873 32977359 PMC7646648

[37] Abdelmottaleb W Salmon JT Quintanilla Rodriguez BS Portillo I Mushiyev S. COVID-19 myopericarditis with pericardial effusion complicated with cardiac tamponade and rhabdomyolysis. Cureus. (2022) 14(7):1. 10.7759/cureus.27291 PMC9403220 36039245

[38] Ammirati E Lupi L Palazzini M Hendren NS Grodin JL Cannistraci CV Prevalence, characteristics, and outcomes of COVID-19–associated acute myocarditis. Circulation. (2022) 145:1123–39. 10.1161/CIRCULATIONAHA.121.056817 35404682 PMC8989611

[39] Kumar K Ratnakumar R Collin SM Berrocal-Almanza LC Ricci P Al-Zubaidy M Chest CT features and functional correlates of COVID-19 at 3 months and 12 months follow-up. Clin Med (Lond). (2023) 23:467–77. 10.7861/clinmed.2023-0059 37775167 PMC10541283

[40] Hastie CE Lowe DJ McAuley A Winter AJ Mills NL Black C Outcomes among confirmed cases and a matched comparison group in the long-COVID in Scotland study. Nat Commun. (2022) 13:5663. 10.1038/s41467-022-33415-5 36224173 PMC9556711

[41] Pérez-González A Araújo-Ameijeiras A Fernández-Villar A Crespo M Poveda E Long COVID in hospitalized and non-hospitalized patients in a large cohort in northwest Spain, a prospective cohort study. Sci Rep. (2022) 12:3369. 10.1038/s41598-022-07414-x 35233035 PMC8888560

[42] Scurati R Papini N Giussani P Alberti G Tringali C The challenge of long COVID-19 management: from disease molecular hallmarks to the proposal of exercise as therapy. Int J Mol Sci. (2022) 23:12311. 10.3390/ijms232012311 36293160 PMC9603679

[43] Altmann DM Whettlock EM Liu S Arachchillage DJ Boyton RJ The immunology of long COVID. Nat Rev Immunol. (2023) 23:618–34. 10.1038/s41577-023-00904-7 37433988

